# Circulating and Salivary Antibodies to *Fusobacterium nucleatum* Are Associated With Cystic Pancreatic Neoplasm Malignancy

**DOI:** 10.3389/fimmu.2020.02003

**Published:** 2020-08-28

**Authors:** Hassan Alkharaan, Liyan Lu, Giorgio Gabarrini, Asif Halimi, Zeeshan Ateeb, Michał J. Sobkowiak, Haleh Davanian, Carlos Fernández Moro, Leif Jansson, Marco Del Chiaro, Volkan Özenci, Margaret Sällberg Chen

**Affiliations:** ^1^Department of Dental Medicine, Karolinska Institutet, Huddinge, Sweden; ^2^College of Dentistry, Prince Sattam Bin Abdulaziz University, Al-Kharj, Saudi Arabia; ^3^Tenth People’s Hospital, Tongji University, Shanghai, China; ^4^Pancreatic Surgery Unit, Division of Surgery, Department of Clinical Science, Intervention and Technology, Karolinska University Hospital, Huddinge, Sweden; ^5^Division of Pathology, Department of Laboratory Medicine, Karolinska Institutet, Huddinge, Sweden; ^6^Department of Clinical Pathology/Cytology, Karolinska University Hospital, Huddinge, Sweden; ^7^Clinic of Endodontics and Periodontology, Eastman Institute Stockholm, Stockholm, Sweden; ^8^Division of Surgical Oncology, Department of Surgery, University of Colorado, Aurora, CO, United States; ^9^Division of Clinical Microbiology, Department of Laboratory Medicine, Karolinska Institutet, Huddinge, Sweden

**Keywords:** pancreatic cancer, pancreatic cyst neoplasm, IPMN, oral commensals, humoral response

## Abstract

**Objectives:**

Intraductal papillary mucinous neoplasms (IPMNs) are cystic precursor lesions to pancreatic cancer. The presence of oral microbes in pancreatic tissue or cyst fluid has been associated with high-grade dysplasia (HGD) and cancer. The present study aims at investigating if humoral immunity to pancreas-associated oral microbes reflects IPMN severity.

**Design:**

Paired plasma (*n* = 109) and saliva (*n* = 65) samples were obtained from IPMN pancreatic cystic tumor cases and controls, for anti-bacterial antibody analysis and DNA quantification by enzyme-linked immunosorbent assay (ELISA) and qPCR, respectively. Tumor severity was graded by histopathology, laboratory, and clinical data. Circulating plasma and salivary antibody reactivity to a pancreas-associated oral microbe panel were measured by ELISA and correlated to tumor severity.

**Results:**

The patient group with high-risk cystic tumors (HGD and/or associated invasive cancer) shows ample circulating IgG reactivity to *Fusobacterium nucleatum* (*F. nucleatum*) but not to *Granulicatella adiacens* (*G. adiacens*), which is independent of the salivary bacteria DNA levels. This group also shows higher salivary IgA reactivity to *F. nucleatum*, Fap2 of *F. nucleatum*, and *Streptococcus gordonii* (*S. gordonii*) compared to low-risk IPMN and controls. The salivary antibody reactivity to *F. nucleatum* and Fap2 are found to be highly correlated, and cross-competition assays further confirm that these antibodies appear cross-reactive.

**Conclusion:**

Our findings indicate that humoral reactivity against pancreas-associated oral microbes may reflect IPMN severity. These findings are beneficial for biomarker development.

## Introduction

Pancreatic cancer (PC) is highly lethal, as the statistics of its incidence rate are closely similar to those of its mortality rate (1). It is the fourth most prevalent cause of cancer mortality in the United States (1), and approximately 330,400 die of PC each year worldwide (2). Yearly incidence rates of PC are dramatically increasing, and PC is expected to be the second top cancer killer before 2030 (3). Up to 90% of PC patients die within 5 years of diagnosis, and more than 50% of them die in the first 6 months after the diagnosis^1,2^. A substantial reason behind the high lethality of this disease lies in the obscure symptoms at its early stages, which result in late stage detection (4). Consequently, early detection of PC is a pivotal step in reducing the PC mortality rate, but much remains to be explored regarding the etiopathology as well as the diagnostic and therapeutic approaches to this cancer.

Pancreatic cystic neoplasms (PCN) became a common type of clinically detected lesion with the current advancement of cross-sectional imaging diagnostic modalities (5–7). One of the most common PCN types is intraductal papillary mucinous neoplasm (IPMN) (8, 9). IPMNs are epithelial neoplastic cysts in the ductal systems of the pancreas, distinguished by the papillary projection of proliferated epithelial cells and mucin secretion that leads to a dilated pancreatic duct (10). IPMNs are characterized by their association with malignancies, as it is believed that the dysplastic pattern of IPMN can progress from low-grade dysplasia (LGD) to high-grade dysplasia (HGD) with the potential to transform into invasive carcinoma (9, 11). The growing incidence of IPMNs and their tendency to progress toward invasive cancer focus great attention and efforts on detection of their benign forms, or LGD, which constitute the majority of cases and do not require surgical intervention (5, 8, 10). Preoperative diagnostic accuracy is still a major challenge that impacts the criteria for surgical resection; hence, the final diagnosis can only be confirmed histologically after operation (5, 10). This raises important issues with over- or under-treatment that could be better addressed by more accurate diagnostic measures.

Several commensal bacteria have been found to play oncogenic roles in different tumors (12), as microbial dysbiosis with markedly increased bacterial abundance is postulated to negatively impact immunity and foster pancreatic tumorigenesis (13). Distinct species of oral bacteria were found to be associated with distal tumor microenvironments in colorectal cancer and, recently, pancreatic neoplasms (14–16). Among them, *Fusobacterium nucleatum*—a non-motile, non-spore forming, gram-negative, opportunistic anaerobic periodontal bacterium—is garnering attention due to its overrepresentation in colon tumor tissues and has been proposed as a potential oncopathogen (14, 15, 17–19). Enrichment of oral commensals, including *F. nucleatum*, in pancreas is noted to correlate with malignancy in pancreas (16, 20). Presently, the role of oral bacteria in relation to pancreatic neoplasms remains unclear, but circulating antibodies against commensal oral bacteria appear elevated in PC and colorectal cancer (21–23). In contrast, relatively less is known about the salivary antibody responses to such bacteria in relation to the stage of PC development.

Consequently, to further investigate the link between *F. nucleatum* and IPMN, we measured the antibodies against *F. nucleatum* and other oral commensals using pre-operation plasma and saliva samples of IPMN patients. The relationship of cystic tumor severity to salivary bacteria levels was also analyzed. Specifically, the aim of this study is to characterize humoral immunity to oral bacteria previously identified in pancreatic microbiome (16), with a focus on *F. nucleatum*.

## Materials and Methods

### Study Population and Sample Collection

Patients with cystic lesions undergoing pancreatic surgery for suspected PC at Karolinska University Hospital, Stockholm, Sweden, were prospectively included in 2017–2019 as participants after signing informed consent forms. The final pancreas diagnosis confirmed by post-operative histology reports further divide the cohort into the following sub-groups: low-grade dysplasia IPMN (LGD-IPMN), high-grade dysplasia IPMN (HGD-IPMN), or IPMN with invasive cancer (Cancer); and non-IPMN. Clinicopathology dta were retrieved from medical journals by physicians. Blood samples were obtained from 109 participants at the day of the surgery, and 65 also donated saliva samples. Additional healthy donors without pancreas diagnoses (*n* = 8) were recruited for clinical oral health examination and saliva donation at the Karolinska University Hospital. Plasma was prepared after centrifugation in K2 EDTA tubes (BD Vacutainer) and Ficoll Paque PLUS (GE Life Sciences) density gradient according to manufacturers’ instructions, then immediately stored at −80°C. Saliva samples were collected the day before the pancreas surgery. The participants were asked to refrain from eating, drinking, smoking, or using oral hygiene products for at least 1 h prior to collection. Expectorated fresh stimulated saliva was collected in sterile 50 mL polyethylene tubes on ice, and thereafter immediately stored at −80°C in 1.5 mL aliquots.

### Periodontal Self-Reported Questionnaire

The questionnaire to evaluate the oral health condition comprised a set of eight closed-ended, self-reported questions identified by the Centers for Disease Control and Prevention (CDC) in collaboration with the American Academy of Periodontology (AAP) (24, 25). These questions were formulated based on the CDC-AAP case definitions for surveillance of periodontitis (26) assessing gum and teeth health, dental treatment history, bone loss, loose teeth, teeth appearance, and usage of dental floss and mouthwash. The questionnaire was distributed prior to surgery to the participants. Data obtained were analyzed to compare the oral health condition between PCN groups.

### Bacterial Strains, Handling, and Inactivation Methods

An array of clinical isolates related to previously reported pancreas microbiome were identified by Matrix-Assisted Laser Desorption/Ionization Time-of-Flight Mass Spectrometry (MALDI-TOF MS). *F. nucleatum* and *Porphyromonas gingivalis* were isolated from clinical blood cultures, identified as in standard clinical routine by MALDI-TOF MS, and were stored at −80°C. The isolates were then thawed and cultured on blood agar in anaerobic milieu 48 h at 37°C. *Streptococcus gordonii*, *Streptococcus anginosus* (*S. anginosus*), *Granulicatella adiacens*, and *Escherichia coli* (*E. coli*) strains were identified by MALDI-TOF MS after having been isolated from the pancreatic cyst fluid of the present patient cohort and then cultured on blood and hematin agar plates. The plates were incubated at 37°C and were examined after 24 h. Glycerol was then added to the liquid cultures (20% final concentration) prior to storage at −80°C. The bacterial pellets were inactivated for enzyme-linked immunosorbent assay (ELISA) analysis by heat inactivation. Isolated bacteria pelleted at 1 × 10E9 CFU were washed twice in PBS and heat-killed at 85°C for 1 h. The B cell mimotope peptide of *F. nucleatum*, Fap2 protein (Fap2), of the sequence TELAYKHYFGT described earlier by prediction analysis using the Immune Epitope Database (IEDB) Analysis Resource (22), was synthesized to a purity of 98% (Genscript, New York, United States). As an alternative to heat inactivation, bacterial pellets were inactivated by being resuspended in 0.5 mL Tris–buffered saline, fixed in 0.5% formalin-PBS overnight at 4°C, and washed three times with PBS.

### ELISA Assays

Heat-inactivated bacteria were prepared as coating antigens to determine antibody reactivity by ELISA. *P. gingivalis*, *S. gordonii*, *S. anginosus*, and *G. adiacens* were used as oral bacterial controls and *E. coli* as a non-oral bacterial control. Indicated bacteria and peptide were resuspended in coating buffer (sodium carbonate buffer 50 mM, pH 9.6) to reach a concentration of 5 × 10E7 CFU/mL and 10 μg/mL, respectively. Afterward, 100 μL were added to each well of a Nunc MaxiSorp™ 96-well ELISA plate (Sigma-Aldrich Sweden AB, Stockholm, Sweden) for overnight coating at 4°C. Wells were washed three times with washing buffer [0.05% Tween-20 (VWR Chemicals, Spånga, Sweden) in PBS]. A buffer of 1% BSA and 2% goat serum (Sigma-Aldrich, G6767) in PBS was used as blocking and dilution buffer. Wells were incubated with the blocking buffer for 1 h at 37°C, and then washed 3 times with the washing buffer. After an initial assay optimization, plasma and saliva samples were diluted with the dilution buffer into 1:300 and 1:16, respectively, then incubated in duplicate wells for 1 h at 37°C on antigen-coated plates. For total IgG and IgA ELISA, samples were diluted 1:125 000 and 1:5000 for plasma and saliva, respectively. Plates were washed as described above and incubated with peroxidase antibody produced in goat anti-Human IgG/IgA (Sigma-Aldrich Sweden AB; diluted 1:10 000) for 1 h at 37°C. After washing, the wells were developed with tetramethylbenzidine substrate (R&D Systems, Minneapolis, Minnesota, United States) for 20 min and stopped immediately with 0.16 M sulfuric acid, and the absorbance was read at 450 nm (Multiskan MS, Thermo Labsystems, Vantaa, Finland). Internal controls consisting of a pool of high-respective low-reactive patient plasma or saliva from HG-IPMN + cancer resp. control group were included in each run. If the OD of internal controls deviated more than 10% from previous runs, the entire plate was repeated. The OD measurements of internal controls were used to calculate the inter/intra-assay coefficient of variability for all runs. Inter-assay coefficient of variability was 8.5 ± 4.5% and 10.4 ± 2.3%, respectively, for plasma and saliva assays, while the intra-assay coefficients of variability were 8 ± 2.3% and 8.7 ± 1%, respectively. The competitive ELISA assay was performed by coating plates with Fap2 peptide overnight. Saliva samples with or without pre-incubation with *F. nucleatum*, Fap2, or the *E. coli* control for 2 h at 37°C were added to the Fap2 peptide-coated plates and developed as above. Total levels of salivary IgA and plasma IgG antibodies were measured using Human IgA/IgG ELISA Kit (Novus Biologicals, Colorado, United States) according to the manufacturer’s instructions.

### Salivary Bacterial DNA Isolation

Microbial DNA was isolated from 200 μL of bacterial culture or saliva using ZymoBIOMICS™ DNA Mini Kit (Zymo Research, Irvine, California, United States) according to the manufacturer’s instructions and eluted into 100 μL of RNAse free water. Isolated DNA was stored at −20°C until use.

### qPCR Quantification of Microbial gDNA Levels

Saliva DNA isolated from 200 μL of saliva were analyzed by qPCR using 7500 Fast Real-Time PCR system (Applied Biosystems, Waltham, Massachusetts, United States). Bacterial gDNA of *F. nucleatum* and *G. adiacens* were used for both standard curves and positive controls, while nuclease-free water and blank PCR Master Mix were used for negative controls. Each sample for *F. nucleatum* analysis included, together with 5 μL of template DNA, 500 mM forward primer: 5′-AGGGTGAACGGCCACAAG-3′, 500 mM reverse primer: 5′-TCTCGGTCCATTGTCCAATATTCC-3′, 200 mM probe: 5′-FAM- ACACGGCCCTTACTCC -TAMRA-3′ (16, 27), and 10.0 μL TaqMan™ Fast Universal PCR Master Mix (ThermoFisher Scientific, Waltham, Massachusetts, United States) and RT-PCR grade water (Invitrogen, Waltham, Massachusetts, United States), for a total reaction volume of 20 μL. The composition of samples for *G. adiacens* analysis differed only in primers and probe: 5′-CAAGCTTCTGCTGATGGATGGA-3′ as forward, 5′-CTC AGGTCGGCTATGCATCAC-3′ as reverse primer, and 5′-FAM-GCTAGTTGGTGAGGTAACGGCTCA-TAMRA-3′ as probe (28). Primers for 16S gene analysis were: Forward 5′-TGGAGCATGTGGTTTAATTCGA-3, and reverse 5′-TGC GGGACTTAACCCAACA-3 primers, as 16S probe was 5′-FAM-CACGAGCTGACGACA[A/G]CCATGCA-TAMRA-3 (29). Samples were heated at 95°C for 10 min, followed by 40 cycles of 95°C for 15 s and 58°C for 30 s.

### Statistical Analysis

All statistical analyses were performed with GraphPad Prism (version 8.00 for Windows, GraphPad Software, La Jolla, CA, United States). Pairwise statistical comparisons between each group were made using Kruskal–Wallis test with Dunn’s multiple comparisons correction for quantitative parameters and Fisher’s exact test for qualitative values. The difference in antibody responses between the groups was analyzed by using Kolmogorov–Smirnov test. The correlation between plasma IgG and salivary IgA reactivity against *F. nucleatum* was examined with Pearson analysis. For the competition assay analysis, Wilcoxon test pairwise statistics was used. The ELISA cut-off values of saliva samples were computed based on the formula described by Frey et al. on healthy controls (30). Values higher than the cut-off were regarded as reactive while those that were lower as non-reactive.

## Results

### Characteristics of the Study Participants

A total of 109 participants with clinical and radiographically confirmed PCN with suspicion of cancer undergoing surgery during 2017–2019 were recruited for the study. Based on final postoperative histopathology reports, the cases were sub-grouped into: HGD-IPMN + Cancer (IPMN with invasive cancer; *n* = 46), LGD-IPMN (*n* = 45), and non-IPMN (*n* = 18; Table 1). This gives a pre-operative diagnosis accuracy at 52, 0, 65% for non-IPMN, LGD-IPMN, and HGD-IPMN + Cancer, respectively, based on comparisons of the pre-operation diagnosis. Moreover, the non-IPMN cases were often younger, and fewer were male or had diabetes. Elevated serum CA19-9 level was found in 55.6% in HGD-IPMN + Cancer group, and 6.7–16.7% in the other groups.

**TABLE 1:** Patient characteristics – plasma cohort.

	**Plasma (*n* = 112)**		
	**Control**	**LG-IPMN**	**HG-IPMN + Cancer**
Parameters	(*n* = 18)	(*n* = 45)	(*n* = 46)
Gender (F:M)	16:2	24:21**Ω	22:24***Ω
Age (years) median (range)	46.5 (30–71)	70 (36–84) ****Ω	73 (47–88) ****Ω
BMI (kg/m^2^) median (range)	27 (20–39)	26.5 (20.4–39)	24.5 (18.4–32.9)
Smoking (%)	25	12	13
Diabetes (%)	0	18	32.6*Ω
CVD (%)	33	20	24
Alcohol (%)	43	47	43
Antibiotic (%; <1 month)	0	0	11
Pre-op. diagnostic accuracy (%)	50	0***ΩΦ	65
S-CA 19-9 (kE/L) median (range)	9 (<1–63)	8.5 (<1–182) ****Φ	51 (<l–5370) *Ω
S-CA 19-9 above normal baseline§ (%)	16.7	6.7 ****Φ	55.6 *Ω
CRP (mg/L) median (range)	1 (1–10)	1 (<1–74)	3 (<1–130)
HbA1c (mmol/mol) median (range)	36.5 (30–50)	39 (30–69)	40 (3–102)
Serum amylase (μkat/L) median (range)	0.36 (<0.13–1.69)	0.46 (<0.13–8.68)*Φ	0.33 (<0.13–4.2)
Albumin (g/L) median (range)	39 (33–42)	37 (31–46) *Φ	36 (16–40) ***Ω

Pairwise statistical comparisons between each group were made using Kruskal–Wallis test with Dunn’s multiple comparisons correction for quantitative parameters and Fisher’s exact test for qualitative values. Bold indicates statistical significance (*p < 0.05, **p < 0.01, ***p**<** 0.001, and ****p < 0.0001). Ω Indicates comparison with control group. Φ indicates comparison with HG + cancer group. §Normal baseline S-Ca 19–9 < 34 kE/L.

### Plasma Reactivities to Oral Commensals Associated With IPMN Cancer Signature

**FIGURE 1:**
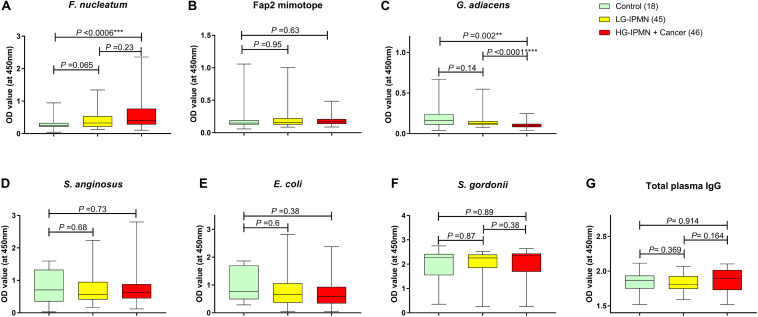
Circulating plasma antibody reactivity to oral commensals identified in pancreatic tumor lesions. Plasma diluent (1/300) from patients with histology verified pancreas tissues: IPMN with low grade dysplasia (LGD-IPMN), IPMN with high grade dysplasia or with invasive cancer (HGD-IPMN + Cancer), or benign tumors/non-IPMN (Control) was tested in duplicates in ELISA plates coated with indicated antigen in **(A–F)** (bacteria: 5 × 10E7 CFU/ml, peptide 10 μg/mL). The total plasma IgG level is shown in **(G)**. Internal control Mean OD absorbance values of duplicates are shown. Statistical analysis was performed using the Kolmogorov–Smirnov test, **p < 0.01, ***p < 0.001, and ****p < 0.0001.

Plasma samples were tested for binding reactivity to *F. nucleatum, G. adiacens, S. gordonii, S. anginosus*, and a Fap2 mimotope of *F. nucleatum* by indirect IgG and IgA ELISA. *E. coli* was included as a non-oral commensal control. In Figure 1, as compared by the absorbance readings, the IgG binding reactivities to *F. nucleatum* in the HGD-IPMN + Cancer group appeared higher than non-IPMN (control group) but not LGD-IPMN group. Reactivities to the Fap2 mimotope of *F. nucleatum*, *S. anginosus*, *S. gordonii*, or *E. coli* did not differ between the groups (Figures 1B–F). But reactivities to *G. adiacens* were noted to be weaker in HGD-IPMN + Cancer than other groups (Figure 1C). Moreover, no significant difference was found when the total plasma IgG levels were compared (Figure 1G). Of note, plasma IgA reactivities were weak in general; no significant differences were noted between the groups, irrespectively of the antigen tested (data not shown).

### Salivary Reactivity to Oral Commensals Associated With IPMN Cancer Signature

**FIGURE 2:**
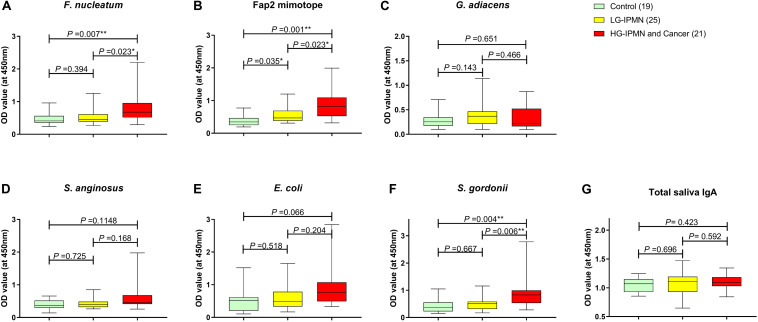
Salivary IgA antibody reactivity to oral commensals quantified by ELISA using saliva diluent (1/16) from indicated patient group. Salivary IgA reactivity to *F. nucleatum*, the Fap2 mimotope, and *S. gordonii* **(A,B,F)** in HG-IPMN/Cancer patients is significantly increased compared to other groups. The differences in antibody levels against bacterial antigens of *G. adiacens, S. anginosus*, and *E. coli* **(C,D,E)** were insignificant between groups. Total plasma IgA level did not differ between the groups **(G)**. Statistical analysis was performed using the Kolmogorov–Smirnov test, *p < 0.05 and **p < 0.01.

Because bacteria of interest here mostly colonize the oral cavity, saliva samples donated by the patient cohort above were tested. To provide a better baseline for saliva tests, orally healthy participants (*n* = 8) without pancreas diagnosis were included in the control group (Table 2). From the total of 65 saliva donors tested, salivary IgA appeared to dominate over IgG (data not shown). In Figure 2, the results indicate that salivary IgA reactivity to *F. nucleatum* in the HGD-IPMN + Cancer group was higher than both LGD-IPMN and control groups. This was shown also for the Fap2 mimotope of *F. nucleatum*, as well as for *S. gordonii* (Figures 2A–F), but not for the other bacteria tested in the panel, and is independent of the total salivary IgA which appear comparable in all three groups. As noted in oral health data, these groups report similar frequency of gum bleeding or untreated gum disease and bone loss. These two groups’ perceptions of own dental health and other oral parameters are also similar (Supplementary Table S1). Moreover, the Fap2-mimotope- or whole bacteria-binding antibody (OD readings) levels when expressed as percentage of reactive or non-reactive saliva samples suggest that 52.4% of HG-IPMN + Cancer group had Fap2 mimotope reactivity in saliva, whilst up to 61.9% had salivary reactivity to any Fap2-mimotope, *F. nucleatum*, or *S. gordonii* antigen (Supplementary Table S2).

**TABLE 2:** Patient characteristics – saliva cohort.

	**Saliva (*n* = 65)**		
**Parameters**	**Control (*n* = 19)**	**LG-IPMN (*n* = 25)**	**HG-IPMN + Cancer (*n* = 21)**
Gender (F:M)	10:9	14:11	10:11
Age (years) median (range)	56 (28–74)	72 (45–87) ***Ω	74 (47–84) ****Ω
BMI (kg/m^2^) median (range)	25 (19.5–39)	26 (21.1–36.2)	27 (18.8–31)
Smoking (%)	21.0	16.0	15.0
Diabetes (%)	15.8	20.0	38.1
CVD (%)	39	68.0	52.4
Alcohol (%)	36.8	48.0	35.0
Antibiotic (%; <1 month)	15.8	20.0	15.0
Pre-op. diagnostic accuracy (%)	36.3γ	0.0 ***ΩΦ	43.3
S-CA 19-9 (kE/L) median (range) Above normal baseline§ (%)	9.4 (3–63)γ 9.1γ	7.6 (2.5–25) **Φ 20.0 *Φ	83 (4–1970) *Ω 57.9 *Ω
CRP (mg/L) median (range)	2 (<1–13)γ	1 (<1–22) *Φ	3.5 (<1–126)
HbA1c (mmol/mol) median (range)	40.5 (30–50)γ	40 (33–62)	42 (28–68)
Serum amylase (μkat/L) median (range)	0.32 (<0.13–0.75)γ	0.42 (<0.13–0.86)	0.4 (<0.13–1.56)
Albumin (g/L) median (range)	39 (34–42)γ	38 (30–42)	36 (16–40) *Ω

Pairwise statistical comparisons between each group were made using Kruskal–Wallis test with Dunn’s multiple comparisons correction for quantitative parameters and Fisher’s exact test for qualitative values. Bold indicates statistical significance (^∗^*p* < 0.05, ^∗∗^*p* < 0.01, ^∗∗∗^*p***<** 0.001, and ^****^p < 0.0001). Ω Indicates comparison with control group. Φ indicates comparison with HG + cancer group. Ɣdata available for 11 non-IPMN cases. §Normal baseline S-Ca 19–9 < 34 kE/L.

### Correlation of *F. nucleatum* Binding to Fap2 Mimotope in Plasma and Saliva, and Cross-Reactivity Validation

**FIGURE 3:**
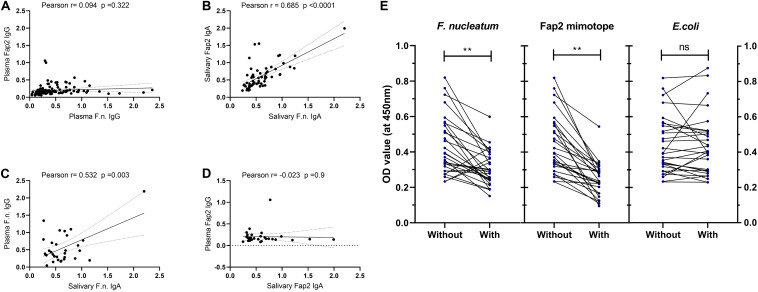
Correlation between circulating plasma **(A)** or salivary **(B)** antibody reactivity to *F. nucleatum* and Fap2 mimotope, or between circulating plasma antibody and salivary antibodies to either of antigen **(C,D)**. Competitive ELISA **(E)** with saliva samples with or without antigen pre-absorption as indicated for 2 h, then subjected to Fap2 mimotope ELISA test. Statistical analysis was performed using two-tailed Pearson correlation test and Wilcoxon test for two related samples. **p < 0.01, ns = not significant.

Because the increased salivary IgA reactivity to *F. nucleatum* and the Fap2 mimotope were noted separately, subsequent correlation analysis was carried out. It could confirm that the two IgA specificities in saliva are clearly correlated unlike that of plasma IgG (Figures 3A,B). The plasma and salivary antibody reactivities to *F. nucleatum*, but not to Fap2, are also significantly correlated (Figures 3C,D). To further examine the specificity interactions of salivary IgA between *F. nucleatum*- and Fap2 mimotope-binding antibodies, a competition ELISA utilizing the mimotope coated plates was performed. A pre-absorption for 2 h at 37°C, either with *F. nucleatum* or the Fap2 mimotope, was enough to reduce the Fap2 mimotope binding efficiency significantly by 24 ± 31% and 40 ± 20%, respectively, but not with *E. coli* (1.7 ± 25%; Figure 3E). Taken together, these results confirm that antibodies to the Fap2 mimotope in saliva cross-react with *F. nucleatum* and may interfere with the antigen binding sites.

### Salivary Bacteria Copy Numbers in Relation to Pancreas Diagnosis

**FIGURE 4:**
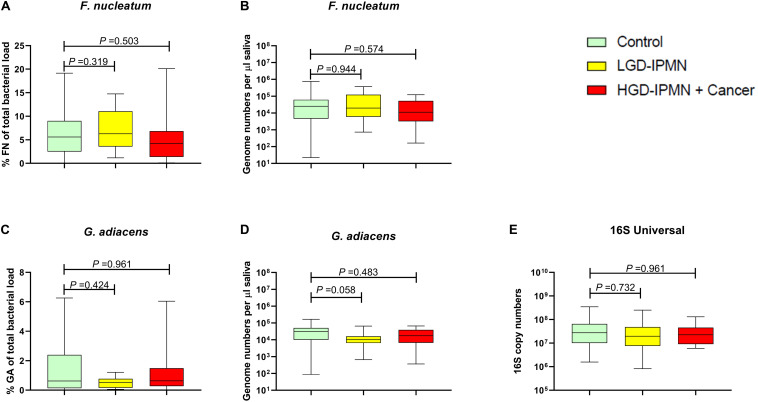
Quantification of *F. nucleatum* and *G. adiancens* genome counts in saliva in patients of indicated diagnose group, expressed as relative of total salivary 16S copy count of the sample **(A,C)**, or as per μl of sample **(B,D)**. Total salivary bacterial 16S genome counts in indicated group **(E)**. Statistical analysis was performed using Mann–Whitney *U* test.

To determine whether *F. nucleatum* and *G. adiacens* salivary antibody levels are attributed to the salivary bacteria, bacterial DNA copy levels were measured by targeted qPCR Taqman assays. Interestingly, the levels of *F. nucleatum and G. adiacens* DNA appear to be relatively comparable in all three groups, regardless of the method used to express them (i.e., as percentage of total salivary 16S copy level or genome copy number per volume saliva; Figures 4A–D). Total salivary bacterial DNA as assessed by universal 16S gene copy levels showed comparable levels among the three groups (Figure 4E). Additionally, correlation analyses showed no significant association between bacterial DNA copy levels to either bacteria antibody reactivity, smoking, or antibiotic use in the past 3 months prior to sample collection (data not shown).

## Discussion

We examined humoral responses to selected oral pathogens that have been previously identified intratumorally in PCN microbiota. Our findings indicate that IPMN cases with high-grade dysplasia or that progressed into invasive cancer have ample antibody reactivities to these oral microbes. In particular, *F. nucleatum* bacteria are recognized by circulating IgG as well as salivary IgA. In this group, salivary IgA reactivity to *F. nucleatum* was found to extend to the Fap2 immunodominant region of *F. nucleatum*. The notable positive correlation between *F. nucleatum* and Fap2 salivary IgA reactivities indicates that the antibodies exist within the same individuals’ saliva. Our data also suggest that they are not only cross-reactive at antigen level but also appear to antagonize, as confirmed by the cross-competition assay. Thus, they should be able to bind and attach to salivary *F. nucleatum* in the oral cavity, where the bacteria normally reside. It is relevant given that Fap2 is the bacterial outer membrane adhesin employed by *F. nucleatum* to bind itself to its target, in the tumor microenvironment it has been shown to impair antitumoral NK cell functions and induce lymphocytic apoptosis through TIGIT and CEACAM1 activation. Moreover, Fap2 is also involved in mediating tumor cells attachment by binding to Gal-GalNAc lectin sugar moiety that is overexpressed on tumor cells (31–34). Although secreted mucosal IgA plays a critical role in host defense against pathogenic bacteria, a recent interesting study suggests that gut microbiota may also utilize IgA for mucosal colonization (35), a mechanism that seems to exist *in vivo* allowing commensal species to exploit IgA specifically to promote its establishment. Surface capsule modified mutants of human commensal *Bacteroides fragilis* were shown to have this attribute and supported host-microbial symbiosis in experimentally mono-colonized mouse models.

Of note, considering that clinical fusobacteria strains naturally lacking Fap2 may exist and Fap2 expression could be abolished by transposon modifications (31, 34), the differential Fap2 salivary IgA reactivities seen here are therefore intriguing. Further investigation into oral colonization of Fap2-overexpressing strains could perhaps shed more light on this. Moreover, in spite of the correlation between plasma and salivary antibody to *F. nucleatum*, the plasma antibody reactivity to Fap2 detected in the HGD-IPMN + Cancer group appears as low as other groups. It is not clear if it may block a Fap2-mediated attachment of *F. nucleatum* in the case of a systemic dissimilation. Furthermore, the HGD-IPMN + Cancer group shows an increased antibody reactivity to *S. gordonii* in saliva, along with a limited antibody reactivity to *G. adiacens* in circulation. Although these bacteria were isolated from pancreas, their potential as an oncopathogen has not been described. However, *S. gordonii* cross-feeding mechanism driving mutualism with *F. nucleatum* (36) could potentially foster a co-existence and perhaps support immunogenicity of *S. gordonii*.

In colorectal cancer, circulating IgG antibodies to *F. nucleatum* and the *Fap2* mimotope were recently reported to be increased (22, 23), but a recent European population study questions the diagnostic potential of *F. nucleatum* proteins in pre-diagnostic serum samples (37). Whether salivary antibody studies could contribute would be interesting to explore. As in colorectal cancer, the oral commensal *F. nucleatum* in pancreatic cystic fluid has been found to associate with pancreatic malignancy (16, 20). Although *F. nucleatum* is more renowned as a periodontal pathogen (38) and periodontal disease might increase the risk for PC (39), patients under periodontal treatment seem not to have elevated *F. nucleatum* antibody levels as compared to periodontally healthy controls (40, 41). In our study, the majority of HGD-IPMN + Cancer patients disclosed no more periodontal conditions than the LGD-IPMN patient group. In spite of this, it is likely that past periodontal insults or dysbiosis are equally important in regulation of immune memory to commensal bacteria. Additional areas to explore include immune memory and tissue resident memory cells (42, 43) that should help gain more understanding into the complex dynamics of immune recognition of cancer-associated commensals in individuals at risk of pancreas malignancies.

Generally, antigens trigger the immune response to secreted antibodies. These humoral antibodies have been widely used in the diagnosis and tumor screening of many diseases. *F. nucleatum* humoral antibodies have been reported in several studies to be significantly increased in patients with Alzheimer’s disease (44), aortic atherosclerosis (45), chronic bronchitis (46), and colorectal cancer (23). Intriguingly, in line with our observations, plasma antibody responses to *F. nucleatum* were not significantly showing a difference between pre-diagnosed PC and matched controls (21). The potential of a saliva IgA assay could therefore be further investigated.

*Fusobacterium nucleatum* has long been considered an opportunistic pathogen, given its frequent isolation and identification in anaerobic samples from patients with different infections. Recently, *F. nucleatum* has garnered much attention in colorectal cancer microbiome studies (47), and an association between the presence of *F. nucleatum* and human colorectal cancer has emerged across the tumor stages. Although well known to oral and medical microbiologists and new studies are revealing the intricate ways in which a bacterium can contribute to the development and spread of colorectal cancer and induce resistance to chemotherapy (48–50), the clinical relevance of *F. nucleatum* in development and prognosis of PC remains to be studied.

Currently, the majority of PCs are diagnosed at a late stage of their natural history when they are symptomatic. Individuals with cystic lesions represent high-risk cohorts which should be entered into surveillance programs, despite the fact that only a subset of them develop PC. Determination of cancer risk biomarkers will require multiple input parameters such as polygenic risk score, BMI, smoking history, as well as other variables (51). Surveillance and diagnosis of asymptomatic PC in longitudinally monitored high-risk cohorts will require biomarkers with discriminating sensitivity and specificity to avoid the risks of under- or over-diagnosis.

The main limitations of this study were the small sample size and having only single blood and saliva measure at one time point. Saliva sampling is presently not a routine element in pancreas oncology programs; therefore, not all patients could donate saliva in this study, though it could be integrated in the near future. However, here the patient subgroups were properly matched with histologically validated LGD-IPMN vs. HGD-IPMN + Cancer cases, which are the two most challenging groups to differentiate in the clinic. Efforts were taken to include an additional baseline control group with bona fide benign pancreas tumors, including paired saliva from orally healthy individuals. Although our results are interesting, and have not been reported earlier to our knowledge, we did not compare the cellular immunity. Intracellular replication is a feature of fusobacteria (52), and this possibility may occur in pancreas tissues; thus, it could be a target for immune killing also in the tumors. Another limitation of this study was the lack of investigation on whether drug use (e.g., antibiotics or NSAID use) may have influenced bacteria antibody levels.

Although our work is restricted to the present microbiota antigen modalities and patient cohort, we believe our results are attractive for the future development of a non-invasive and affordable diagnostic biomarker for patients at risk of PC, for accurate identification and selection of a suitable intervention to approach a cancer cure.

## Data Availability Statement

The raw data supporting the conclusions of this article will be made available by the authors, without undue reservation.

## Ethics Statement

The studies involving human participants were reviewed and approved by Regionala Etikprövningsnämnden i Stockholm. The patients/participants provided their written informed consent to participate in this study.

## Author Contributions

MSC, VÖ, MD, and HA: study design. HA, LL, and GG: sample collection and processing. AH, ZA, CF, HA, and LJ: clinical data collection and interpretation. HA, GG, LL, and VÖ: laboratory work. HA, GG, MJS, and MSC: data analysis and statistics. HA, MSC, GG, MJS, and HD: manuscript preparation. HA, LL, GG, AH, ZA, MJS, HD, CF, LJ, MD, VÖ, and MSC: approval of final draft submission. All authors contributed to the article and approved the submitted version.

## Conflict of Interest

The authors declare that the research was conducted in the absence of any commercial or financial relationships that could be construed as a potential conflict of interest.
