# Supplementary material for: Circulating and Salivary Antibodies to Fusobacterium nucleatum Are Associated With Cystic Pancreatic Neoplasm Malignancy
Source: Front Immunol. 2020 Aug 28;11:2003. doi: 10.3389/fimmu.2020.02003 (PMC7484485; doi:10.3389/fimmu.2020.02003)
Supplement: Supplementary file 1 [file Data_Sheet_1.PDF]

Supplementary Material

Table S1

	Saliva (n=56)		
**Parameters**	**Control (n=19)**	**LG-IPMN (n=22)**	**HG-IPMN + Cancer (n=15)**
Teeth brushing (≥2 /Day) (%)	84.2	86.4	87.5
Gum bleeding ≥ once/week (%)	36.8	27.3	16.7
Vegetarian (%)	0	0	0
Food intake (#daily frequency) (%)	3 (2-5)	3 (2-6)	3 (3 - 4)
Sweets consumption (per week) median (range)	3 (0-14)	2 (0-21)	2 (0-7)
Flow rate (mL/min) median (range)	1.8 (1-6)	1.6 (0.6-2.8)	1.9 (0.6-3)
Have gum disease (%)	10	19	22
Excellent/Very good/Good health of teeth and gums (%)	91	79	73
Treatment for gum disease (%)	31	57	38
Loose teeth (%)	0	29	9.10
Bone loss (%)	10	11	18
Tooth does not look right (%)	10	10	10
Use dental floss (%)	70	75	70
Use mouthwash (%)	16	35	18

Pairwise comparison between the groups using logistic regression and age. the questions included as independent variables showed no significant associations between group and the answers of the questionnaire using using Kruskal-Wallis test for quantitative data and Fisher's exact test for qualitative values.

Table S2

		Salivary IgA	
		**Reactive**	**Non-reactive**
**Fap2 mimotope (Fap2)**	Control	0.0%	100.0%
	LG-IPMN	16.0%	84.0%
	HG-IPMN + Cancer	52.4%	47.6%
F. nucleatum (F.n.)	Control	0.0%	100.0%
	LG-IPMN	4.0%	96.0%
	HG-IPMN + Cancer	38.1%	61.9%
S. gordonii (S.g.)	Control	0.0%	100.0%
	LG-IPMN	4.0%	96.0%
	HG-IPMN + Cancer	28.6%	71.4%
**Fap2 or *F.n* or *S.g.***	Control	0.0%	100.0%
	LG-IPMN	12.0%	88.0%
	HG-IPMN + Cancer	61.9%	38.1%

Salivary reactivity to Fap2-mimotope- or indicated whole bacteria of IPMN patient groups and controls. The ELISA cut-off values were computed on OD values of healthy controls (30). Values higher than the cut-off were regarded as reactive while those that were lower as non-reactive.
